# The brainstem connectome database

**DOI:** 10.1038/s41597-022-01219-3

**Published:** 2022-04-12

**Authors:** Oliver Schmitt, Peter Eipert, Frauke Ruß, Julia Beier, Kanar Kadir, Anja Horn

**Affiliations:** 1 Dep. of Anatomy, University of Rostock, Rostock, Germany; 2 MSH Medical School Hamburg, University of Applied Sciences and Medical University, Hamburg, Germany; 3 Institute of Anatomy and Cell Biology I, Ludwig-Maximilians-Universität München, Munich, Germany

**Keywords:** Computational neuroscience, Computational biology and bioinformatics

## Abstract

Connectivity data of the nervous system and subdivisions, such as the brainstem, cerebral cortex and subcortical nuclei, are necessary to understand connectional structures, predict effects of connectional disorders and simulate network dynamics. For that purpose, a database was built and analyzed which comprises all known directed and weighted connections within the rat brainstem. A longterm metastudy of original research publications describing tract tracing results form the foundation of the brainstem connectome (BC) database which can be analyzed directly in the framework *neuroVIISAS*. The BC database can be accessed directly by connectivity tables, a web-based tool and the framework. Analysis of global and local network properties, a motif analysis, and a community analysis of the brainstem connectome provides insight into its network organization. For example, we found that BC is a scale-free network with a small-world connectivity. The Louvain modularity and weighted stochastic block matching resulted in partially matching of functions and connectivity. BC modeling was performed to demonstrate signal propagation through the somatosensory pathway which is affected in Multiple sclerosis.

## Abstract (summary)

**Table:** 

Measurement(s)	brainstem
Technology Type(s)	tract tracing metastudy
Factor Type(s)	brain region
Sample Characteristic - Organism	Rattus rattus
Sample Characteristic - Environment	Experimental setup
Sample Characteristic - Location	Germany

## Background & Summary

Neuronal connections between regions of the nervous system enable the transmission of dynamic signals. Information about the existence, absence or lack of data on neuronal connections and their features is important for realistic modeling of signal transfer in connectomes. Conceiving the effects of connectome lesions requires comprehensive and accurate connectivity data as well as properly modelled dynamic processes. The global and local impacts of lesions caused by stroke or neurodegenerative disorders like Multiple sclerosis, Parkinson and Alzheimer disease allow their simulation and dynamical analysis in such accurate structural connectomes^1^.

Until now, connectomes^2^ of a few species have been elaborated^3–13^. However, a microconnectome at the synaptic level is available only for the nematode *Caenorhabditis elegans*^12^. The microconnectome of the ultrastructural volume of the *Drosophila melanogaster* is still not on-hand^13^. More realistic connectomes are generated by accumulating tract tracing (TT) data (TTD). Since 1971, neuronal connections of strains of laboratory rats were investigated extensively and repeatedly by applying TT techniques^14^ (Table 1). These precise neuroanatomical TT data are collated in meta-studies and considered as gold standard for comparisons with tractographic data^15,16^. However, the interpretation and translation of descriptions of projections and overlapping terminologies may lead to variability of TT connections^17^. A longterm project^18,19^ systematically collated and curated neuronal connectivity data and connectional features of original TT research publications^11,17,18^. Here, a new brainstem connectome database is introduced comprising every known connection of every region of the brainstem. In the brainstem connectome (BC) project, *all hiterto known* neuronal connections of the brain stem nuclei complexes of the adult rat were collated. This BC database can be queried through a web interface for all regions reported in original research publications.

**Table 1:** Some tract-tracer substances and factors which are axonally transported or propagated by diffusion.

Tracer family	Examples	Dir	Vel	Pub
Proteins	Horseradish peroxidase (HRP)	R/A	F	^141^
	Albumin			^14^
	Immunoglobulin M (IgM)	R		^142^
Anorganic fluorochromes	Fast Blue (FB)	R	M	^143–145^
	Diamidino yellow (DY)	R		^146^
	Fluoro-gold (FG)	R		^147^
Dextranes	Fluoro-Ruby (FR)	A/R	M	^148,149^
	Biotinylated dextran amine (BDA)	A/R		^150^
Lectines	Wheat germ agglutinin (WGA; WGA-HRP)	R/A	F	^151^
	Bandeiraea simplicifolia isolectin B4 (IB4)	A		^152^
	Phaseolus vulgaris-leucoagglutinin (PHA-L)	A		^153,154^
Beads	Latex microspheres	R	F	^155^
	Cholera toxin B-gold	R		^156^
	Wheat germ agglutinin-apoHRP gold	R		^157^
Bacterial toxins	Tetanus toxin fragment C (BIIb)	R/A	F	^158,159^
	Botulinum toxin A (BoTu)	R/A		^160^
	Cholera toxin B fragment (CTB)	R/A		^161^
Growth factors	Nerve growth factor (NGF)	R	F	^162,163^
	Glial cell-derived neurotrophic factor (GDNF)	A		^164^
	Ciliary neurotrophic factor (CNTF)	R		^165^
Amino acids	3H-Leucin	A	F/S	^166,167^
	3H-Prolin	A	F/S	^168^
Vit. biotin and L-lysine	Biocytin	A	F	^169^
Carbocyanine dyes	DiI	A/R	S	^170^
	DiO	A/R	S	^171,172^

S: slow, M: medium, F: fast, A: anterograde, R: retrograde, A/R: bilateral transport, Dir: axonal transport direction, Vel: transport velocity, Vit: vitamin, Pub: Publication, DiI: 1,1′-diocta decyl-3,3,3′,3′-tetramethylindodicarbocyanine perchlorate, DiO: 3,3′-dioctadecyloxacarbocyanine perchlorate.

Compared to other metastudy connectome databases and subsystems of connectomes^20–26^, a significant feature of the BC database is the preservation of precise neuroanatomical information. Same connections within different animals and/or reports are always recorded. Thus, accumulations of same observations in different animals are available for statistical analysis and thresholding of high consensus connections and rare observations of neuronal connections. This permits the calculation of *observation scores* and estimating reliability^10,17^. For an overview of all features regarding the data sources, collation, curation and validation we refer to^17,18^. Moreover, the BC database is linked with six secondary databases (bibtex bibliographies^27^, NeuroLex^28^, BrainInfo^29^, Lockard^30^, NeuroElectro^31^, Swanson Terminology^32^). An export function in *neuroVIISAS* allows the generation of *mysql* databases which are accessible in the web^33^.

The brainstem is a structurally and functionally complex control center of the rat nervous system. A structural and visual analysis of connectomes should be performed^34^ before they are characterized by dynamic modeling. So far, the BC has not been characterized quantitatively in terms of connectome analysis. Here, such an analysis of intrinsic hierarchical connectivity of the BC is performed with regard to the structural network organization as well as some functional properties.

**Fig. 1:**
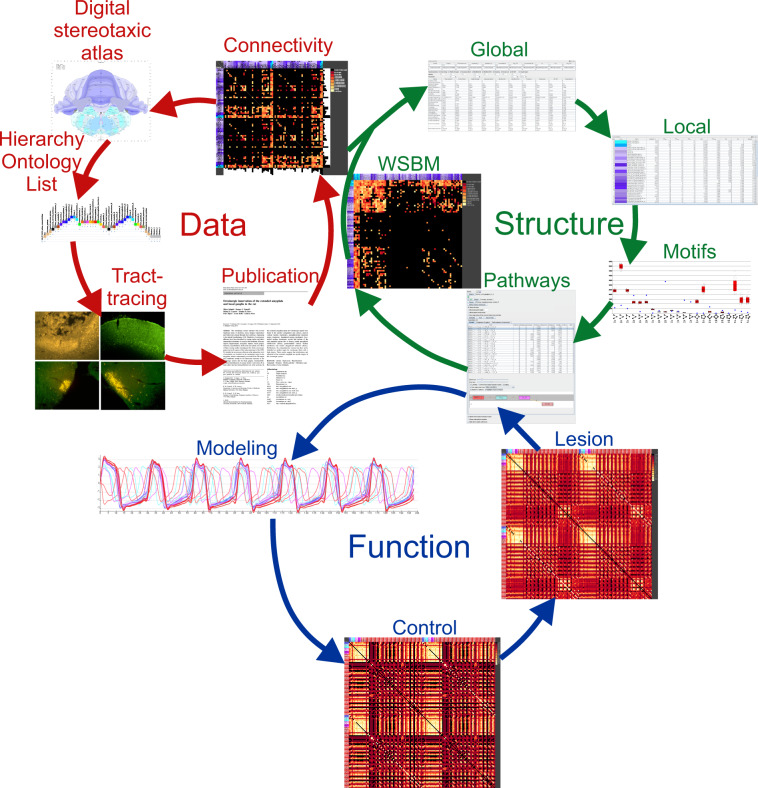
Schematic outline of the brainstem connectome generation and simulations in the *neuroVIISAS* framework. Data generation starts with hypothesis of possible connections using stereotaxic atlases and concepts of knowledge (ontologies). Then TT experiments are performed and connections are published. Original research publications were evaluated and connections described herein were collated and imported into the rat connectome database to build the connectivity of adjacency matrices. These are starting points for global and local as well as motif network analysis. *neuroVIISAS* provides tools to investigate pathways and community detection algorithms like weighted stochastic block matching (WSBM), Louvain modularity and spectral graph analysis among oth-ers. The weights of a pathway can be reduced to model demyelination disorders (lesion) which can be compared with control coactivation matrices derived from excitatory FitzHugh Nagumo network propagation models.

Hitherto, neuroinformatic toolboxes are used to analyze and visualize connectomes^35–40^. In addition, generic network analysis environments are available^41–43^. The *neuroVIISAS* (**neuro V**sualization, **I**magemapping, **I**nformation **S**ystem for **A**nalysis and **S**imulation) framework allows the analysis of connectivity categories (structural, functional, visual) (Fig. 1) of macroscale to microscale as well as hierarchical connectomes. *neuroVIISAS* is a generic and platform-independent framework that links ontologies, digital atlases, connectomics, and simulations of whole-brain network dynamics. By using this framework, connectivity data obtained from tractographic, TT and serial block face scanning microscopy can be investigated under different aspects like structural organization, functional properties and dynamic features (Fig. 1). Recently, a differential connectomics module for pairwise network comparison has expanded the analytical capabilities of the *neuroVIISAS* framework^44–46^. The framework allows working with tables of (non-)weighted and/or (non-)directed connections as well as with hierarchical or neuroontological (non-)weighted and/or (non-)directed connections among hierarchically organized superregions and subregions. So far, connectomes of different species and of highly diverse structural organisations (unilateral, bilateral, weighted, binary, directed, non-directed) can be directly loaded with *neuroVIISAS* and are available in proper project file formats (https://neuroviisas.med.uni-rostock.de/otherConnectomes/otherConnectomes.shtml).

The main purpose of this contribution is to introduce the first BC database. A second issue is the structural and functional analysis of the BC connectome using the *neuroVIISAS* framework. Because the BC is embedded in a complex subcortical and cortical connectome it will be analyzed with regard to extrinsic connectivity as well. This allows the investigation of the somatosensory pathway from the dorsal root ganglia through the brainstem and diencephalon to the somatosensory cortex. Among other pathways and functional systems the somatosensory pathway is significantly affected in Multiple sclerosis through $CD{4}^{+}$ T-helper cell and $CD{9}^{+}$ cytotoxic T cell dysregulation^47–50^. A further objective of this investigion is to elucidate how signal propagation within the somatosensory pathway is affected by changing connectivity weights in comparison with a demyelination process in MS.

## Methods

### Tract-tracing data

Collating information of neuronal connections between pairs of neuroanatomically defined regions can be performed by manually reading out data from original research publications which describe the anterograde and retrograde transport of tract-tracing substances. The original research publications of this study were filtered from Pubmed (https://www.ncbi.nlm.nih.gov), GoogleScholar (https://scholar.google.com/), Scopus (https://www.scopus.com) and Web of Science (http://apps.webofknowledge.com/). After matching the 4 database queries, the references were imported into Jabref (http://jabref.sourceforge.net/) to obtain a unique and flexible bibtex style. Up to now, the database has been continuously updated. Added references were immediately put into the pipeline of systematic curation. The database is available at https://neuroviisas.med.uni-rostock.de/references.html or https://neuroviisas.med.uni-rostock.de/daten/references.bib and can be downloaded from^51^ as well. Such a metastudy approach to generate connectomes is well established and has been performed successfully in ferret, avian, macaque, cat and rat^4,5,7,8,10,11^.

Automatic recognition might represent another possibility to readout neuronal connections from original research publications. However, the algorithmic analysis of the semantic structure of the connection description still produces too many false positive and false negative identifications. Moreover, the identification of heterogeneous presentations of connectivity data in the form of texts, tables and figures is far beyond the capabilities of text spotting systems (generative adversarial networks, deep learning, semantic-based text recognition)^52–55^.

High-throughput TT of whole brains is a great advance for generating the connectome of the mouse brain^3,56,57^, however, the spatial resolution and the parcellation of neuroanatomical regions are limited. In addition, tractographic analyses of diffusion tensor imaging data allow the computation of adjacency matrices^58–61^. A great advantage of DTI connectomes is the measurement *in vivo* and straightforward application to genetically and experimentally (Multiple sclerosis, Parkinson, depression, stroke models) modified animals. The interregional connections do possess spatial orientations, even though they are not directed. Further methods which generate connectomes at different scales of resolution (microconnectome, mesoconnectome, macroconnectome) are described in review articles^62,63^.

The approach applied here for generating a connectome is considered to be a metastudy^64^ or retrospective study as performed by many groups in the field^7,8,20–26,52,65–75^. In order to prevent skewing of original research literature in terms of structures that have been studied, all 7867 publications which applied TT techniques (Table 1) were used for collation of connectivity data. TT publications were excluded which describe connectivity in prenatal rats, genetically modified rats (knock-in, knock-out, RNA silencing) or experimentally modified rats (neurodegenerative disease models, stroke models, intoxications etc.). The advantage of this procedure is that afferent and efferent connections of the BS were gathered which are incidentally observed in TT experiments in other parts of the brain.

Further bias which possibly skew the BC data may arise from TT experiments of preferentially investigated functional systems that are abundantly affected in neurological disorders. Considerably more connectivity information may originate from these preferential investigations than from neuroanatomical TT studies independent of hypothesis-driven neurological disorder studies.

Finally, we tested the hypothesis that bias may arise from the volume of BS regions because small regions are less studied or shape a larger variability of identifying tracers. There was evidence that a small linear positive correlation of c=0.2 exists between the volume and the number of different original research publications. However, the quite small locus coeruleus region (0,079 *mm*^2^) has been identified in more the 750 research publications. Thus, besides the size of a region, the functional relevance and importance in neurological disorders may skew the distribution of connectivity information in a metastudy connectome as well.

In most stereotaxic tract-tracing studies, tracers are used that are actively transported anterogradely to axonal terminals or retrogradely to perikarya (Table 1). Once the tracers have reached their target areas, they can be visualized in most methods either by immunohistochemical or enzymatic substrate conversion. These chromogens, detectable in histological sections, provide the underlying information for the connectional data, which are specified and documented at three different levels of accuracy. After successful visualization in histological sections or mostly systematic section series with specific section distances, visualized tracers are localized or assigned to specific areas. At level 1, this assignment "tracer to area" is done in the original publications purely descriptively, in schematic block diagrams or semi-schematic figures. Most often, at level 2, medium precision documentation is presented by means of symbolic (*, **, *** or -, +, ++, +++) or semiquantitative (0, 1, 2, 3: no connection, light, moderate, strong) data of the observed neuronal connections in the form of text or tables. These ordinal measures represent an estimate of connection densities. Level 3 is based on either stereological quantification of perikarya labeled by tract tracing or densitometric quantification of labeled axonal terminals. Semiquantitative documentation of tract-tracing observations is most commonly used in the literature. So far, the semiquantitative values can not be normalized between studies and thus comparability is limited. The stability of the underlying chromogen, which makes the neuronal connections visible, measurable and estimable, is relatively high. Observation and discovery of the tracer as well as its assignment to areas also depends on the experience of the investigator. When analyzing the connectivity data, we reliably recognize these different categories of neural connection descriptions and are able to code the information about connections and weights as follows.

All neuronal connections are encoded by ranked qualitative connections weights from the primary research literature. The most frequent categories are $x$: very few [0.5], few [1], few to moderate [1.5], moderate [2], moderate to strong [2.5], strong [3] and very strong [4]. The primary research publications describing results of experimental TT suggest that a realistic scale for ranked qualitative values is exponential rather than linear. A 10^5^ exponential scale was applied for such values in the cerebral cortex of the macaque^76^. Here, a 10^4^ exponential scale ($f(x)$) fit the rat data better^77–81^:

$$f(x)=1{0}^{\left(-\frac{16}{49}\cdot {\left(x-4\right)}^{2}\right)}$$

## Data Records

All data files and software are hosted at figshare^51^. Two different types of data records are relevant for the work with BC connectome data in *neuroVIISAS*. A complex data record (CDR: bc.brain. A direct download is possible from https://neuroviisas.med.uni-rostock.de/bc.brain) allows to specify different analyses of BC. The straightforward data record (SDR: bc.csv) is a simple list of all intrinsic neuronal connections of the brainstem nuclei.

The rat brainstem connectome data are available by querying the publications, longnames or shortnames of regions of the brainstem through a web interface of *neuroVIISAS* (https://neuroviisas.med.uni-rostock.de/connectome/index.php)^33^. This connectome resource has been indexed by FAIRsharing (bsg-d001343)^82^.

In addition, the adjacency matrix is downloadable as a connection list (*bc.csv*)^51^ (straightforward data record: SDR) and a *neuroVIISAS* project data file (*bc.brain*)^51^. The latter contains all data records of the brainstem regions where each connection consists of 20 data fields (complex data record: CDR):

- *Description of source*
  A short description of source region features with regard to tracer visualization.
- **Source**
  The unique abbreviation of a source region.
- *Description of target*
  A short description of target region features with regard to tracer visualization.
- **Target**
  The unique abbreviation of a target region.
- **Weight**
  Ordinal weights $$w=\{-\,3,-\,2,-\,1,-\,0.5,0,0.5,1,1.5,2,2.5,3,4\}$$
- LessIpsi
  Indicates if tracing is stronger ipsilateral than contralateral $i > c$ or contralateral than ipsilateral $c > i$.
- **Reference**
  The ID of the publication which is included in a *.bibtex file (e.g., “Lu:2019a”).
- **Tracer**
  The unique abbreviation of a tracer that was used to detect the connection described by the data record.
- *Case*
  An abbreviation for an animal or experiment in which a connection has been detected. The abbreviations are those which are used in the publication.
- *Animal*
  Strain of experimental animal.
- *Labeling*
- *Soma*
  Specification of region containing perikarya which are the source of a connection.
- *SomaNote*
  Expression of proteins, modulators, transmitters and receptors of a region where a connection begins.
- **Laterality**
  The connection links ipsilateral, contralateral or unilateral regions:
  - IPSI: ipsilateral
  - CONTRA: contralateral
  - LL: unilateral left side of brain, body, organization
  - RR: unilateral right side of brain, body, organization
  - LR: contralateral connection from left to right
  - RL: contralateral connection from right to left
- *Terminal*
  Specification of region containing the axon terminals which are the target of a connection.
- *TerminalNote*
  Expression of proteins, modulators, transmitters and receptors of a region where a connection terminates.
- *Terminalic*
  Specification of axonal termination with regard to laterality.
- *TransportDirection*
  The direction in which a tracer was transported in the axoplasm with possible values:
  - r: retrograde
  - a: anterograde
  - a/r: retrograde and anterograde
  - ta: anterograde transsynaptic transport with unknown intermediate regions
  - tr: retrograde transsynaptic transport with unknown intermediate regions
  - tma: anterograde transsynaptic transport within a monosynaptic connection
  - tmr: retrograde transsynaptic transport within a monosynaptic connection
- *Modality*
  The modality designates an entity of a *connectional system*:
  - P: pathway connection
  - C: collateral connection: singular monosynaptic connection
- *Page*
  The page on which the connection has been described.
- *Annotation*
  Comments about the connection.
- *Collator*
  Surname of the person who generated the data record.
- *Date*
  The date when the data record was generated.
- *Sex*
  The sex of the experimental animal: m: male, f: female, m, f: male or female, no entry: unknown sex.
  - f: female
  - m: male
  - m, f: male or female: unknown sex

The data fields **Source**, **Target**, **Weight**, **Publication** and **Laterality** are mandatory for importing new connection records into the BC project in the *neuroVIISAS* framework. However, data records can be re-defined or custom-built. The removal of data fields within a project file is not supported. Appending a new field to a record is compatible with importing new fields and adding them to the data structure with a certain number of data fields. Thus, importing new connectional data structures is possible.

This data record is designed for large and complex connectome projects. However, the relatively short list of neuronal connections of the SDR in form of tabulator separated *Source*, *Target*, *Weight* data fields in a csv text file is easier to access (an example of a strongly connected random network can be downloaded^51^). The only requirement for such a light import of connectivity data is the definition of a project (root of hierarchy or list of regions). Features like laterality and publication links are not defined by importing links and weights, only. Nevertheless, they can be defined following the import of the text file.

The BC connectome dataset is openly available for download from figshare^51^. Available files for download are the following:

- bc.brain: the complete brainstem connectome with longnames, shortnames, laterality information, weights and links to references.bib.
- bc.csv: the weighted connectivity data with region codes for loading in spreadsheet software (11717 connections). It is the version of the straightforward data record (SDR).
- scn.csv: Strongly connected directed random network (null model) as a csv text file.
- neuroVIISAS_windows-x64_1_4_2_4.exe: the analysis framework for analyzing the BC connectome dataset for a MS windows operating system.
- neuroVIISAS_unix_1_4_2_4.sh: the analysis framework for analyzing the BC connectome dataset for a Linux operating system.
- neuroVIISAS_macos_1_4_2_4_Folder.dmg: the analysis framework for analyzing the BC connectome dataset for a iOS system.
- references.bib: Bibtex database representing all references of original research papers which were used to build the BC connectome.

Since new concepts of neuronal, neuroendocrine, neuromodular and temporary dynamic connections are developed, the data structure allows extensions of field containment. Thus, it is possible to add new types or concepts of synaptic connections to the item *Modality*, such as

- Neuronal electrochemical synapse^83^.
- Temporary synapse: Within adult hippocampal neurogenesis new excitatory granule cells are generated in the dentate gyrus. Their axons form the mossy fiber tract that links the dentate gyrus to CA3^84–86^.
- Tripartite synapse (gliotransmission)^87^.
- Quadpartite synapse^88^.
- Enteroendocrine-vagal-synapse^89^.

So far, three types of connection modalities have been defined in the database. The classical monosynaptic electrochemical connection is defined by leaving the entry of the *Modality* field blank. Because very many collateral observations were made by injecting two or multiple tracers and because all collaterals belong to a particular neuron, they can be considered as a *connectional entity*. Another modality of *connectional entity* is a pathway which is sometimes described in virus TT studies. This concept of administrating complex modalities of connections is proposed here for the first time. Of course, analysis of *collateral connection entities* and/or *pathway connection entities* is supported by the *neuroVIISAS* framework.

In the CDR type of database, connections documented in different publications are clearly distinguishable by their *Publication* ID. Therefore, the import of one table of data, which may contain connections of different publications, is a common way of appending new data to a *neuroVIISAS* project.

A further novelty of the connectome database in general, and of the BC database in particular, is the realization of accumulating the same connections with different features. Different features could be different experimental cases within the same publication or the same connections described in different publications. Interestingly, we found different weight values and comments of the same connections in different publications. Adding these complete raw data to the project database in *neuroVIISAS* allows us to identify and to filter high and low conformity as well as difference of neuronal connections.

### Connectome databases in *neuroVIISAS*

Project files are compressed databases which are loaded in the framework. Various project files can be imported in particular project frames allowing the comparison of different connectomes^46^ for *differential connectome analyses*.

In the simplest case, the mandatory data of a project is just one region. More generally, such a singular “region” is a root node to which lists of regions can be mounted or a hierarchy of regions (typically neuroanatomical regions) can be generated.

A hierarchy of regions can be organized by introducing a spatial ontology or a neuroontology which defines the spatial and topographic relations between distinct regions especially when subregions (children) are branching from superior regions (parents). However, such an ontological definition of a hierarchically organized terminology is not mandatory for a connectome analysis.

Sets of tables (attribute tables) are customizable for different connectome projects. The tables are linked with nodes of the hierarchy. The set of tables in the BC consists of *Chemoarchitectonics* subtables, *Electrophysiology* subtables, *Quantitative information* subtables and *Special information* subtables (*Definition* table for a region, *Function* table for a region, *Cell population* characterization table and a *Commentaries* table).

Each region of the hierarchy can be related to a contour or a closed polygon overlaid on an atlas image, MRI section, microscopic section and other types of image data. The image data are organized as a navigable stack (jumping, scrolling, selecting, searching for image and image contents). If image stacks are imported that need to be defined in a particular reference space like stereotaxic atlases, such a space can be defined even if images are not equidistant within one axis. For the following connectome analysis of the BC, the atlas images of the rat brain are not necessary.

External databases in the framework which are not directly linked with a region represent further knowledge resources. A region of a project hierarchy can be searched directly through all external databases (Brainfo, Lockard, NeuroElectro, Neurolex, Swanson). The import of new databases is possible to enrich the knowledge resources.

Bibtex files contain all references of connectome projects. Specifying a bibtex file in a project, will load it automatically after the following start of the framework. Then it is possible to obtain reference information for each connection. Because pdf locations can be linked with bibtex records, a documented neuronal connection can be found in an original research publication by directly loading the publication into a pdf viewer.

The information environment for documentation and coding of raw data is available in an *Edit connection* frame. It contains a *Trace code* table where each tracer (viral, non viral, passive diffusion tracers) is defined.

## Technical Validation

Two options are used to test the reliability of the connectivity data of this metastudy. First, a collator-independent option compares neuronal connections documented in the original research literature. This approach enables filtering most consensus observations (raw data reliability). Because descriptions of neuronal connectivity observations in original research publications can be complex and sometimes ambiguous, a second option compares neuronal connections in the same original research publication by different collators (collator data reliability).

The different nomenclatures and new definitions of sub-regions are met by the extensible and editable hierarchy of regions in neuroVIISAS. Furthermore, more varying nomenclatures (variants concept) can be selected in the same connectome dataset, depending on the intention of the evaluator. These and other methods for flexibly handling of competing and partially incompatible nomenclatures have been described in detail elsewhere^18^.

Tracer substances can also be taken up by axons-on-passage or fibers-of-passage around an injection site and lead to nonspecific labeling of target regions^23,81,90^. This nonspecific mechanism represents the most important source of error in the interpretation of tract-tracing experiments. Furthermore, different application methods (bolus, pressure, intermittent, iontophoretic and its parameters, gel-foam) of the tracers contribute to the observational variability of neuronal projections. The concentrations of the tracer substances and the applied volumes as well as the exact time course of the stereotactic application can also influence the distribution, uptake and transport in the neuronal compartment. Farther, variability may occur between the sexes, ages, and lineages of the laboratory rats used (Wistar, Sprague-Dawley, Fischer-344, Wistar Kyoto rat, Long Evans hooded rat, Osborn-Mendel). Finally, the variability of survival times after tracer application and the distances of target areas observed in different studies may be related to the semiquantitative weighting of neuronal connections. In the course of our very extensive metastudy, we found that the documentations of experimental conditions was inconsistent. Therefore, we collected exactly the data that are most frequently reported in the studies. And it is on this basis that we will estimate the reliability and observation scores in the following.

To appraise the *raw data reliability* of the neuronal connections, an estimation of the data reliability was performed^91^ by computing an *observation score* $O$. A large observation score of a connection indicates that the probability of the real biological existence of this neuronal connection is large. A requirement for computing $O$ is that most original research publications describing TT results are gathered. As a consequence, the number of observations of all known connections of a connectome is larger than the number of connections. The observation score of a neuronal connection is estimated by adding *reliability weights*. Reliability weights (Table 2) are declared for

- types of tracer transport directions (anterograde, retrograde) (*t* weight of the transport direction of a tracer) and
- the weight or strength of a neuronal connection (*w* weight of the connection strength).

**Table 2:** Reliability weights used for estimating the reliability parameter $o$.

Variable	Case	Value
t	a/r	0.25
t	r	0.5
t	a	0.5
t	r+a/r	0.7
t	a+a/r	0.7
t	a+r	1.0
t	a+r+a/r	1.0
w	−3.0 unknown	0.7
w	−2.0 fibers of passage	0.0
w	−1.0 not clear	0.8
w	−0.5 exists	0.9
w	0.0 not present	−1.0
w	0.5 very light	1.0
w	1.0 light / sparse	1.0
w	1.5 light / moderate	1.0
w	2.0 moderate / dense	1.0
w	2.5 moderate / strong	1.0
w	3.0 strong	1.0
w	4.0 very strong	1.0

Value: reliability weight of connection strength, t: variable of reliability weight for transport directions of tracers, w- variable of reliability weight for strengths of connections, a: anterograde tracer transport, r: retrograde tracer transport, a/r: bidirectional tracer transport. a+r+a/r means that a connection has been proved by an anterograde, a retrograde and a bidirectional transported tracer.

The reliability $O$ is given by:

1 $$O=| \left(\sum {w}_{+}\right)+{t}_{+}+\left(\sum {w}_{-}\right)-{t}_{-}| $$

Multiple observations of the same connection between identical regions are frequently documented in different as well as in the same original research publication in different animals (cases). These multiple connection data are available in the BS database and are used to calculate sums of *w* of case-based and non-case-based experimental observations. If a neuronal connection was observed only by a retrograde *or* anterograde method, then a smaller *t *= 0.5 is assigned than for an observation through an anterograde *and* retrograde method in two independent experiments (*t* = 1). An anterograde and retrograde tracer transport (bilateral: a/r) is allowed in this weighting scheme (Table 2) as well. The subscripts − and + of the variables indicate an observation of an existing connection (${w}_{+}$, ${t}_{+}$) or an explicit description that a connection does not exist (${w}_{-}$, ${t}_{-}$). Hence, explicitly not existent connections are weighted by negative reliability weights. The number of observations of a specific connection is added up ($\sum w$). The value of *t* is calculated by identifying different directions (anterograde, retrograde) of tracer transports within all connections that were added up. For instance, a particular neuronal monosynaptic connection that was observed in 10 different TT experiments by applying anterogradely transported tracers obtains a *w*=10 and a *t* = 0.5 ($O=10.5$). If the connection observed in a total of 10 times was found by using 7 anterograde and 3 retrograde tracers, then *t* = 1 ($O=11$). Another allocation of $o$ parameters may be that a connection is detected 9 times ($\sum {w}_{+}=9$) with 7 retrograde and 2 anterograde tracers (${t}_{+}=1$) and in addition, this particular connection was not found in one experiment ($\sum {w}_{-}=-1$) with an anterograde tracer (${t}_{-}=0.5$), then $O=8.5$. If there are many descriptions in publications that a specific connection does not exist, it gets a strong negative observation score. Many descriptions of the same types of observations of a particular neuronal connection emerge as a consensus observation with a large $O$ value irrespective of whether a connection exists or not. Finally, these scores can be presented in a *reliability matrix*. It should be emphasized that the observation score is defined for the data of a connection between a specific source or efferent region and a target or afferent region. Hence, it is not a score for *biological connections* but rather for the underlying *data of the connections*.

The *inter-rater variation* of a rat connectome metastudy has been evaluated systematically^17^. In that study neuronal connectivity data for three blinded collators were compared. It was found that the variability of the interpretation of neuronal connections from TT original publications is $\le 6{\rm{ \% }}$ (100%: identical lists of neuronal connections of all three raters).

**Fig. 2:**
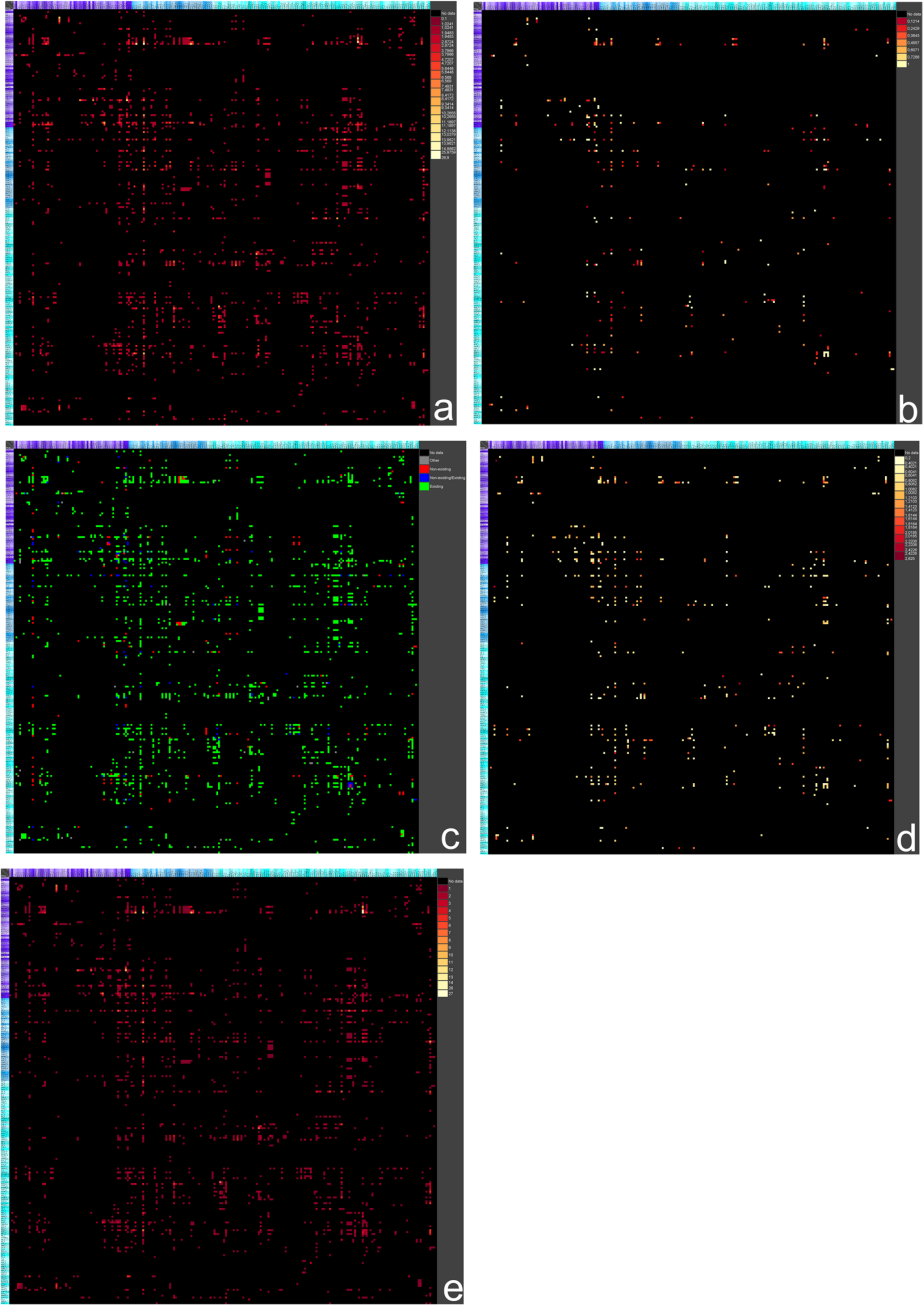
Overview of ipsilateral BS matrices. The magnification of all regions with longnames, shortnames and color codes is shown in table 2.1 of the tutorial. (**a**) Observation score matrix. (**b**) Discrepancy matrix. (**c**) Exist non-exist matrix. (**d**) Variation of weights matrix. (**e**) Number of publications matrix.

By determining the observation score for each observed connection of the unilateral BS *adjacency matrix*, an *observation score matrix* $O$ can be computed (Fig. 2a). Furthermore, the discrepancies of all observed connections can be determined (Fig. 2b). A discrepancy of a connection is maximal (1) if a connection has been documented in an original research publication and the same connection has been explicitly indicated as a non-existent connection in another original research publication. The set of weights is ${w}_{e}$ and for any connection or edge $e=\{{w}_{{e}_{1}},\ldots ,{w}_{{e}_{n}}\}$ was used. The discrepancy of an edge ${d}_{e}$ is given by

$${d}_{e}=\left\{\begin{array}{l}0,{\rm{if}}\forall i,j\,:{w}_{{e}_{i}}={w}_{{e}_{j}}\;i,j\in \{1,\ldots ,n\}\\ 1,{\rm{if}}\exists i\,:{w}_{{e}_{i}} > 0\wedge \exists j\,:{w}_{{e}_{j}}=0\;i,j\in \{1,\ldots ,n\}\\ 0.85\times \frac{{w}_{{e}_{max}}-{w}_{{e}_{min}}}{{w}_{max}-{w}_{min}},{\rm{else}}\end{array}\right.$$

${w}_{max}$ is the maximal possible weight (4.0), while ${w}_{min}$ is the minimal possible weight (0.5). The factor 0.85 is used to scale the fraction to prevent it from becoming 1, the same value for a discrepant result. Hence, the maximum discrepancy of 1 can be distinguished if $\frac{{w}_{{e}_{max}}-{w}_{{e}_{min}}}{{w}_{max}-{w}_{min}}$ becomes 1 as well.

To directly recognize contradictions of observed or not observed neuronal connections, the non-exist - exist matrix can be computed (Fig. 2c). The non-exist - exist matrix ${\bf{E}}$ displays neuronal connections which are described by positive statements (exist), and descriptions of explicitly non-existent connections as well as some observations which find a connection whereas some others do not confirm such a connection.

The non-exist exist attributes of a connection are defined as

$$e{x}_{e}=\left\{\begin{array}{ll}0,{\rm{if}}\forall i\in \{1,\ldots ,n\}\,:{w}_{{e}_{i}}=0 & ( \mbox{''} {\rm{non}}{\rm{ \mbox{-} }}{\rm{exist}}\mbox{''})\\ 1,{\rm{if}}\exists i\;{w}_{{e}_{i}} > 0\wedge \exists j\,:{w}_{{e}_{j}}=0 & ( \mbox{''} {\rm{non}}{\rm{ \mbox{-} }}{\rm{exist}}\,{\rm{and}}\,{\rm{exist}}\mbox{''})\\ 2,{\rm{if}}\forall i\in \{1,\ldots ,n\}\,:{w}_{{e}_{i}} > 0 & ( \mbox{''} {\rm{exist}}\mbox{''})\end{array}\right.$$

And finally, the variability of ordinally scaled weights, which are used in the original research publications as an estimation of axonal density, is quantified by the standard deviation of transformed weights as shown in Fig. 2d. A particular connection that is documented in distinct original research publications with varying strengths can be directly identified by a measure of weight or strength variation. If the variation in the weights of a connection is small, then the average of connection weights can be considered as a more reliable strength than that of a large variation.

Since connectivity data of all original research publications of the BS have been made available, a matrix indicating the number of publications describing each connection can be computed as well. A large number of original research publications of a particular connection means that the real biological existence of this connection is more probable than an observation documented in only one original research publication (Fig. 2e).

## Usage Notes

The data set of the BS connectome is loaded as an *bc.brain* project file^51^ into a *neuroVIISAS* installation on Linux, Windows or iOS. The*.brain* file of a *neuroVIISAS* project contains the compressed data. It is composed of project specific data files. It includes the VTK surface reconstruction of the stereotactic atlas regions and a serialization of the Java object structure of the project with the hierarchy of the regions, the neuronal connections, the contours of the regions and the coordinate systems. The data structure is generalized in such a way that diverse biomedical network data, such as protein interactions from proteomic studies, protein interactions of the SARS-CoV-2 RNA virus, to give only a few examples from other knowledge categories, can be studied in one and the same software infrastructure. Serialization is done using the ObjectOutput stream of Java, which has the advantage of storing and loading a complex data structure quickly and easily.

**Fig. 3:**
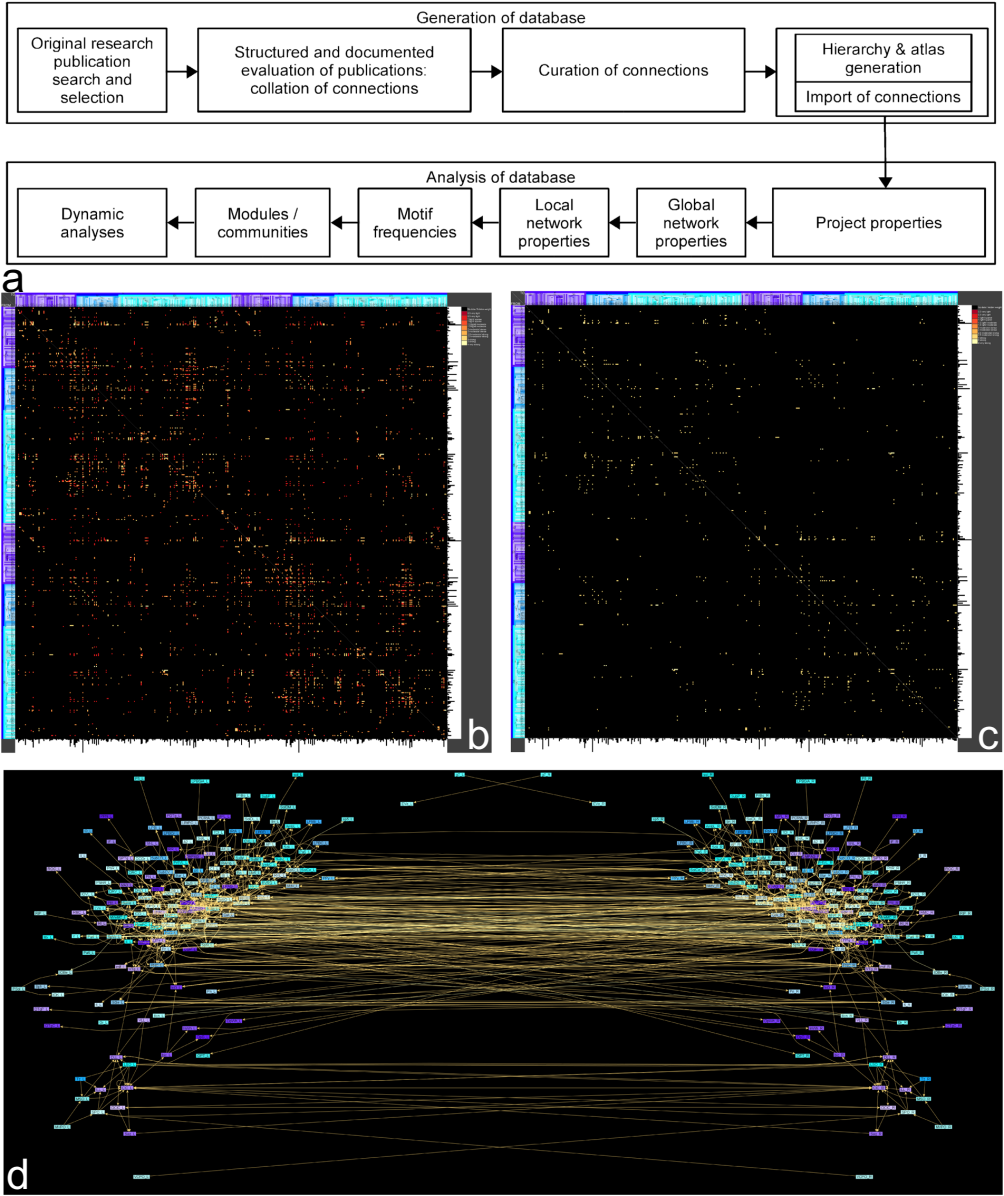
Workflow and visualization of the bilateral BC. (**a**) Workflow of database generation, stepwise data accumulation and data analysis, respectively. (**b**) Overview of bilateral weighted and directed BC. (**c**) Filtered connections with weights $\ge$ 3. (**d**) Bilateral visualization for regions with connection weights $\ge$ 3.

The principal steps (Fig. 3a) for analyzing the BS connectome data start with a general project analysis. Thereafter, an adjacency matrix must be specified to perform a global connectome characterization and a local network analysis. Thereupon, special structural analyses like motif investigations and communication detections may follow. After gaining an overview of the structural features of the connectome, it is far easier to define a simulation of a dynamic process across a principal pathway and signal propagation analysis. In the following, brief instructions are given to assist with the reuse of the BC data, describing important steps in greater detail. After the installation of *neuroVIISAS*, the neuroVIISAS.jar program file can be started by clicking on the *neuroVIISAS* desktop icon (Windows), run.bat (Windows) or run.sh (Linux) batch files. The batch files are located in the installation directories of *neuroVIISAS*. If necessary, the -Xss parameter (stack size) can be increased (“24M“ is a very large value for *neuroVIISAS* project files) by editing the batch files. In the case of starting *neuroVIISAS* directly with a click on the neuroVIISAS.jar file in the installation directory, an error message may appear following loading of the bc.brain project file because the memory and stack size parameters from the batch files are not used. The tutorial provides more information^51^.

The bc.brain project file is loaded by clicking on the entry File in the menu bar of the main window. For this, Open project has to be selected in the pull down menu. Because different links and paths are defined for different projects, the path to the references.bib and to the pdf document directory cannot be found by the newly installed *neuroVIISAS*. If the message window “Document path was not found” appears, then the button Skip should be clicked. The path to the database of references (references.bib) which is also available in^51^ can be set by click on Settings in the menu bar of the main window and then on Change project settings in the pull down menu. Clicking on the button Choose new bibtex file defines the path to the references.bib. Then, it is possible to determine the associated publication for each connection. If this path definition is not performed, the connectome analysis can be done as well without links to references. After successful loading, a project window in the left part of the main window opens. The root node of the BS hierarchy is “13/12/2019_bc_connectome” and the child node of the root node is “Rat”. A double click on the nodes extends the hierarchy. Now it is possible to use all resources for analyzing, visualizing and simulation. The analysis window will be opened by clicking in the menu bar on Analysis and then in the pull down menu on Advanced connectivity analysis. Now the BS hierarchy must be extended to compute the adjacency matrix by pressing the “+” key nine times. Subsequently the Enter key can be pressed and the adjacency matrix in the default view (number of edges) is computed. After this basic selection of regions of the BS connectome, further analyses can be done. If a matrix, table or computational analysis has been selected, it is necessary to press the Refresh button at the lower right corner of the analysis window.

In addition, connectomes of selected regions can be exported into other formats to be loaded, e.g., in Matlab for analyses with the Brain Connectivity Toolbox (BCT)^92^ or DynamicBC^93^. More information on specific functions can be found in the entry Help in the menu bar of the main and analysis windows.

### Project overview

After loading the BC dataset (*bc.brain*), the user assigns the original research report database (*reference.bib*) (Setting menu $\to$ Change project settings $\to$ Choose new bibtex file $\to$ path to reference.bib). Subsequently, the general project statistic shows a quantitative overview of the BC database (Analysis $\to$ Project statistics). 1133 original research publications describe the intrinsic BS connections. A total of 6730 experimental observations provide details about the neuronal connection. The bilateral tree of sub- and superregions consists of 766 nodes. 465 regions are leaves of the region hierarchy. In the whole tree connections between all BS leaves and superregions in both hemispheres are 16306. 2628 reciprocal connections exist in the bilateral BS connectome. The bilateral BC connectome contains 95 collateral entity connections and 84 transsynaptic connection entities. The frequency of weighted connections is light (4242), strong (3400) and moderate (2729).

### Global connectome analysis

The Analysis menu and item Advanced connectivity analysis allow analyzing and performing simulations of the BC dataset. The analysis windows shows 5 frames: Triangle hierarchy, Info, Mini View, Tree hierarchy and Adjacency Matrix. Initially, the Triangle hierarchy frame displays the root node of the hierarchy of regions. The hierarchy expands level by level using the “+” key. Pressing the button Refresh at the lower right bottom of the window or the “Enter” key computes the adjacency matrix. An adjacency matrix contains the information of connections between any pair of regions of a network or connectome. The “Settings” button at the upper right of the adjacency matrix window (beside the two funnel (used for data filtering) buttons) allows a display of the Average weight / Most frequent weight in the weighted adjacency matrix. This specification of the adjacency matrix determines the data for the following network and statistical analyses. The Tab Global parameters opens a table view of global parameters of the specified connectome. Global connectome parameters describe quantitative features of the connectome like how many connections are building the connectome or how many connections are reciprocal. By selecting parameters of interest or unchecking complex ones, like the *reciprocity*, reduces the computing time. This can be done by pressing the “Settings” button at the upper right of the window (there are 4 buttons for the operations of the window layout and one button for the settings with a mouse over function) which contains the global parameter tables. By exporting the computation of different edge (neuronal connections) and node (region) preserving randomization models to a SLURM script (slurm workload manager^94^), the connectome analysis is possible by parallel computation on a computing cluster. 444 regions (222 left and 222 right hemispheric regions) linked by 4785 weighted and directed neuronal connections constitute the bilateral BS connectome. These 444 regions correspond to the distinct regions of the stereotaxic atlas of the rat brain^95^. However, 14 regions are not intrinsically connected. 630 connections are reciprocal. The line density (density of a connectome is the ratio of the number of connections to the number of possible connections in a connectome node) is 2% and the average degree is 19. The degree of a region is the number of connections that are connected to that region and the average degree is the average of all region degrees. The function average path length (the average shortest path length or characteristic path length) calculates the shortest path between all pairs of regions, and computes the average over all paths of the length from that. The BC has an average path length of 2.931. The clustering coefficient of a region is the ratio of existing connections connecting a region’s neighbors to each other to the maximum possible number of such connections. The clustering coefficient for the entire connectome is the average of the clustering coefficients of all the regions of the BC. A high clustering coefficient for a connectome is another indication of a small world. The average cluster coefficient of the BC is 0.2813. The trade off between high local clustering and short path length is the small-worldness. A small average shortest path length and a large clustering coefficient characterize a small world network. The small-worldness is relatively large with 10.9 and the modularity is 0.33. The modularity is a measure of the structure of connectomes. It quantifies the strength of division of a connectome into modules or sets of regions. The error Δ from a scale-free distribution of connections is low (0.5). If distributions of the number of connections of regions (degrees) follow a power law, then it is a scale-free network-free network. The error Δ indicates the differences in the distribution of connectional regions of the BC connectome to the distribution following the power law. A small error Δ indicates a large similarity of the BC and a scale-free network. By comparing 8 different edge- and node-preserving random networks, Watts-Strogatz and rewiring models turned out to be most similar to the BC connectome with regard to other random models. The difference of the Watts-Strogatz networks and the BC connectome indicates a specific connectional organization of the BC connectome.

By filtering the connectome for connection weights $\ge 3$, 1032 connections remain (Fig. 3c). Even after filtering and condensing strong edges of the BC, the connectivity of intrinsically strongly connected BS regions remains complex.

### Local connectome analysis

The computation of local network parameters works in the manner described for the global connectome analysis. The Tab Local parameters opens an empty table where parameters can be specified through the “Settings” button. The *Shapley index* and average values of dynamic simulations ($AvgSEREx$, $AvgRDEx$, $AvgFHNEx$, $FHNSpikes$, $AvgHREx$) are computationally complex. The settings list (upper right button of the local parameter table window) allows unchecking these local parameters. The 444 regions of the bilateral BC are ranked by computing 23 among 54 local network parameters (e.g., $Degre{e}_{all}$, $ClusterCoefficient$, $Katz-status-index$^96,97^, $Excentricity$, $Betweeness$, $Shapley-index$^98^). It turns out that the pedunculopontine tegmental nucleus, laterodorsal tegmental nucleus, median raphe nucleus and raphe magnus nucleus have the largest ranks in the bilateral intrinsic BC connectome. Average large ranks shape strong connectivity ($Degre{e}_{all}$) at strategic positions ($Katz-status-index,Shapley-index$). It is now clear that these regions are most important with regard to network integrity of the bilateral BC. This is also confirmed by vulnerability analysis (results are not shown)^99^.

### Motif analysis

**Fig. 4:**
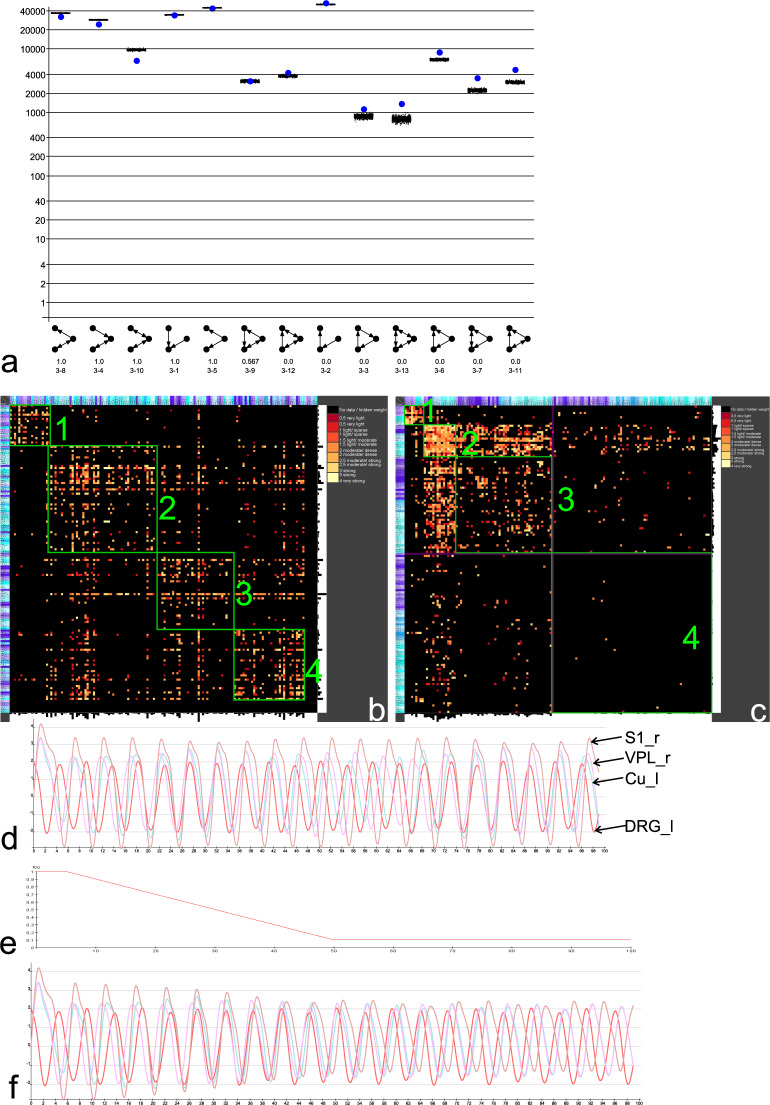
Motif, modularity and dynamic analyses. (**a**) Motif analysis of 13 directed 3 node motifs. The motifs on the x-axis were sorted by the z-values. Blue dots indicate the frequency of motifs in the empirical BS. Black dots indicate frequencies of 1000 edge and node preserving randomization. Y-axes (frequencies) is logarithmically scaled. (**b**) Consensus clustering (10000 iterations) of Louvain modularity with $\gamma =1.0$ of the unilateral BC. 4 modules along the main diagonal were highlighted. (**c**) Weighted stochastic block matching of the unilateral BC (10000 iterations). 3 modules around the main diagonal are clearly visible. The 4th module contains sporadic connections, only. (**d**) FHN simulation with initial condition >0 for left dorsal root ganglia (DRG_l) (red). Magenta curve: Right ventroposterolateral thalamic nucleus (VPL_r), Brown: Right primary somatosensory cortex (S1_r), Turquoise: Left cuneate nucleus (Cu_l). (**e**) Modulation function for connection weights. (**f**) Decrease in membrane potentials of primary somatosensory cortex activation.

Selecting the Tab Motifs initializes the motif analysis. The Motifs generator creates 13 3-node subgraphs. Isomorphism search (Statistics search) determines the frequency of all 13 motifs. The isomorphism search counts these 13 directed 3 node motifs^100,101^ in the bilateral BS connectome and in 1000 edge- and node-preserving Erdös-Rényi randomizations (Fig. 4a). The chain motif 3-02 has the largest frequency. In other studies we have observed that the circular motif 3-07 occurs less frequent in empirical networks than in random networks. Interestingly, the circular motif 3-07 occurs more often in the BC than in 1000 simulations to a slight but significant extent/degree. Notably, more complex motifs (reciprocal edges) have significantly larger frequencies than the simple convergent, divergent or chain motifs.

### Community structure

Before community analysis is available, it is necessary to define a *variant class* in the main window. It allows a rearrangement of regions by the community detection approach. Again, in the Analysis menu, the item Advanced connectivity analysis is selected and the adjacency matrix must be specified for community analysis. The menu Other offers the Hierarchical clustering functions used in the following.

The community structure of the unilateral intrinsic BC (bc.brain project file) with 222 nodes and log transformed weighted 1645 edges was analyzed. The community detection *Louvain modularity analysis* (LMA)^102^ using a $\gamma =1$ and 10000 iterations for consensus clustering^103,104^ determines 4 modules. These 4 modules have some functional preferences (Fig. 4b):

**Module 1 (Olivar):** lateral lemniscus nuclei, para-, peripheral and superior olivar nuclei and nucleus of the trapezoid body

**Module 2 (Vegetative, sensoric):** Ambiguus nucleus, solitary system, gracile nucleus, cuneate nucleus

**Module 3 (Reticular, oculomotoric):** Mesencephalic reticular formation, oculomotor nuclei

**Module 4 (Mesencephalic, motoric):** Mesencephalic nuclei, pontine nuclei, tegmental nuclei

The weighted stochastic block (WSBM) modeling approach using 10000 iterations^105–108^ (Fig. 4c) computes somewhat other modules. The WSBM generates the following 4 modules:

**Module 1 (Collicular, olivar):** Lateral lemniscus nuclei, inferior colliculus nuclei, para-, periolivary and superior olivary nuclei

**Module 2 (Reticular, oculomotor):** Locus coeruleus, reticular formation nuclei, raphe nuclei, pretectal oculomotor nuclei, facial nucleus

**Module 3 (Mesencephalic, motoric, sensoric):** Mesencephalic nuclei, periaqueductal nuclei, noradrenergic cell groups A5 and A7, cuneate nucleus, gracile nucleus, inferior olive

**Module 4 (Mixed-sparse):** Ambiguus nuclei, C2, C3 cell groups, trapezoid nuclei, subcoeruleus nuclei

The modules 1 to 3 of WSBM possess denser connections than module 4. Such a distribution of dense connections and a large sparsely connected module are a typical *core-periphery organization*^105^.

Grouping by consensus clustering of LMA and by WSBM sorts the regions of the adjacency matrix with respect to the density of their connections. Thus, if a group of regions is found to be more strongly connected to each other than to regions of one or more other groups, then this group is called a module. The intrinsic connectivity architecture is thus analyzed to maximize common neural connections within a module. It is important to emphasize that only ipsi- and contralateral directed neuronal connections are used to calculate the composition of the groups.

The module 1 is composed of regions that are primarily components of the auditory system. These include the nuclei of the lateral lemniscus, which contain neurons that are a major component of the ascending auditory pathway and include both monaural and binaural cell groups. They are a major source of input to the inferior colliculus. The superior olivary complex is a collection of smaller nuclei that are important for the ascending and descending auditory pathways. The trapezoid body or ventral acoustic striata forms a part of the auditory pathway that originates from the anterior cochlear nucleus and crosses the side before termination in the superior olivary nucleus. Thus, these intensely neuronally connected regions represent the functional core of the first module. Connectivity here seems to be closely correlated with the same to very similar auditory subfunctions of separable nuclear regions.

Module 2 was assigned to the nucleus ambiguus, the nucleus of the solitary tract (solitary system), the nucleus gracilis, and the nucleus cuneatus, among others. The nucleus ambiguus represents a group of large motor neurons in the depth of the medullary reticular formation. These neuron groups innervate muscles of the soft palate, pharynx and larynx (swallowing). Furthermore, preganglionic parasympathetic motor neurons are present that innervate postganglionic parasympathetic neurons (ganglia) of the heart cardioinhibitory. Thus, ipsilateral branchial efferent motor fibers of the vagus nerve originate from the nucleus ambiguus and terminate laryngeally, pharyngeally, or in the soft palate. In addition, fibers extend from the nucleus ambiguus via the glossopharyngeal nerve to the stylopharyngeus muscle.

In contrast to the motor ambiguus system, the solitarius system processes sensory signals from the facial, glossopharyngeal and vagus nerves. The ambiguus system projects to the reticular formation, parasympathetic preganglionic neurons, hypothalamus (paraventricular nucleus) and thalamus, forming circuits important for processing information for autonomic regulation. Functionally important is the transmission of taste information of the facial nerve via the chorda tympani, the glossopharyngeal nerve and the vagus nerve. Another quality of information of this system comes from chemoreceptors and mechanoreceptors of the general visceral pathway of the carotid body via the glossopharyngeal nerve and from the aortic bodies and sinoatrial node via the vagus nerve. Finally, the nucleus ambiguus chemically and mechanically receives information from the heart, lung, airways, gastrointestinal system and many other organs.

The sensory and motor nucleus complexes of module 2 functionally combine to provide vital reflexes such as the gag reflex or pharyngeal reflex. The assignment of two distinct categories of neuronal functions sensoric vs. motoric in the same module speaks to a circuit concept in which different neuronal processing components must necessarily be arranged to provide integration of neuronal signals that allow organisms to adapt to changing environmental conditions.

Module 3 is composed of the reticular formation and oculomotor core areas among other structural and functional subsystems. An integral part of the reticular formation system is the raphe system with a multitude of important sub-functions such as the suppression of pain reaction (descending pain modulation) and in the broadest sense the control of spinal cord activity. But also the regulation of the sleep-wake cycle, autonomic activity, reproductive behavior and neuroendrine control are important functional components of the medullary and mesencephalic reticular system. The connectivity of the reticular system and the oculomotor nuclei have been assigned to module 3 and this may indicate that ascending reticular activation (ARAS) or CNS arousal system is coupled with the oculomotor system to trigger visual attention at the brainstem level.

Module 4 includes nuclear areas of the mesencephalon, pontine and tegmental nuclei and therefore appears functionally inhomogeneous mainly because corticocerebellar projections are switched in the pons. However the pons can be subdivided into a basal pontine nuclei complex and a reticulotegmental part, so that here too, in addition to a connectional relationship, there is also a functional relationship of the pontine complex to tegmental nuclei composed of the laterodorsal tegmental nucleus, the pedunculopontine nucleus (pedunculopontine tegmental nucleus), the rostromedial tegmental nucleus, and the tegmental pontine reticular nucleus. Functionally, a trigger and activation function of the cortico-cerebello-thalamo-cortical pathway is also suggested here. The biological significance of the modules is to intertwine similar and dissimilar functions by means of neuronal connectivity in such a way that polysynaptic reflexes and complex behavioral patterns can be adapted to changing environmental conditions.

We are aware that the grouping is not perfect and reflects each type of functional component of the brainstem. These include the vestibulo-spinal system, the brainstem respiratory circuits and the control system of the lower urinary tract, which requires carful coordination by neural pathways in the spinal cord to control conscious micturition. One explanation for the incomplete grouping could be the high connectional density to extrinsic regions of the connectome.

The 4 modules calculated by the consensus clustering of the *Louvain modularity analysis* and the 4 modules of the WSBM show obvious differences. The arrangements of BS connectome regions show a *mixed topology*^105^. WSBM arranges regions with functional similarities within same modules. Furthermore, WSBM identifies a core-periphery structure of densely interconnected regions in the first and second module.

### Propagation in a BS connectome coupled FitzHugh-Nagumo simulation

Structural data on neuronal networks in the form of systematically collated connections are an important basis alongside dynamic models and the functional properties of these connections. The importance of dynamic modeling was motivated in detail in^109^. Computational modeling of dynamic processes provides important information about the properties of connectomes and networks. In this respect, different approaches can be applied depending on the questions at hand such as top-down modeling to study the structure-functions relationships of neural circuits or bottom-up modeling to reconstruct a neural system in detail in terms of a reverse engineering approach^110,111^. However, developing a realistic model of brainstem functionality and studying its dynamic properties is problematic for the following reasons.

Immunohistochemical demonstration of marker enzymes of neurotransmitter and neuromodulator metabolism are also detected in various tract-tracing publications. Where this information is available, it has been recorded by us. We have also made this information available in the bc.csv file. Unfortunately, there are also many tract-tracing studies that have other objectives and do not provide information on inhibitory or excitatory connectivity. Even if evidence on inhibitory and excitatory components of microcircuits is available in publications, electrical synapses thus gap junctions and gliotransmission may also modulate excitatory and inhibitory signaling that have not been studied in the same publications. In addition, antagonistic effects of the same neurotransmitter on different postsynaptic receptors such as dopamine 1 receptors and dopamine 2 receptors must be considered. It follows that even if the neurotransmitter-neuromodulator pattern of interconnected regions is known, data on the receptor composition of postsynaptic targets are lacking. All in all, the necessary data are incomplete to perform realistic dynamic modeling of excitatory and inhibitory neuron populations of the brainstem connectome based on neurotransmitter measurement data. Therefore, we did not intend to perform a realistic simulation in the sense of a reverse engineering bottom-up approach. Due to insufficient neuroanatomical data, we implemented another accepted methodology in *neuroVIISAS* to study the flow of information in directed and weighted connectomes. Dynamic models can be used in *neuroVIISAS* based on coupled oscillators (Kuramoto, Stuart-Landau, Chen, van der Pol and many more)^112–117^. Different neural mass models are also available in *neuroVIISAS*^118–121^. Population models can be applied to simulation engine *NEST* in directed and weighted connectomes via a Python interface^122^ which is available as well in *neuroVIISAS*. Several studies investigate connectome and network dynamics using point neuron models such as FitzHugh-Nagumo (FHN)^123–128^, Morris-Lecar^129^, Hodgkin-Huxley^130,131^ and others. Here, we utilized the coupled excitatory FHN model to the directed and weighted brainstem connectome because it is well characterized and can be efficiently computed^123,132–140^. Coupled FHN models are widely accepted to characterize network models and connectomes in terms of their dyamic properties (coherence) as well as to understand the relationship between network topologies and activation patterns. Therefore, the FHN model was also applied to the brainstem connectome for an exemplary application.

The connections between dorsal root ganglia (DRG), ipsilateral brain stem regions, contralateral thalamus (VPL: ventral posterolateral thalamic nucleus) and the contralateral target region primary somatosensory cortex (S1) couple the excitatory FitzHugh-Nagumo (FHN) model neurons^123–127^. For this adaption of the BC connectome the database *bcFHN.brain* was used which is available at figshare. The menu Analysis panel opens a *Simulation* sub-menu with different classes of dynamic models like the FHN-simulation. The Settings button displays modeling parameters. This model uses the direction of connections and the log transformed weights. In a first simulation, the initial conditions are related to DRG_l and connection weights are not modulated. Fig. 4d documents a stable oscillation with weak phase shifts. This pattern of oscillations is still visible by repeating the simulation. In the following, this oscillation pattern is compared with the pattern that emerges when the weights of the connections are changed.

A linear decrease in connections weights shapes the change of weights in terms of a demyelination process (Fig. 4e). The FHN model uses the same parameters like those in the control simulation (Fig. 4e). The oscillations of the non-modified BC are directly compared with the selective weight-modifications BC. It turns out that especially the target region of the somatosensory pathway, which is the right hemispheric primary somatosensory cortex (S1_r), displays a relatively stronger decrease in membrane potentials than Cu_l and VPL_r do (Fig. 4f). The structural change appears to lead to a change of dynamics. The linear decrease in connection weights of the first neuron of the somatosensory pathway (all other weights were not modified) in the BC connectome (all regions and connections were left in place) causes a change of oscillations of the S1 target region relatively (contralateral) far away from the structural deficit in the spinal cord. Further work is needed to determine the interference of signals through multiple inputs and outputs of regions along the somatosensory pathway through the brainstem connectome.

## Data Availability

The code written in JAVA for the generation of the BC database and the analysis of the connectome including the database is available at https://neuroviisas.med.uni-rostock.de/neuroviisas.shtml.
